# Supplementary material for: Human GBP1 is a microbe‐specific gatekeeper of macrophage apoptosis and pyroptosis
Source: EMBO J. 2019 Jun 3;38(13):e100926. doi: 10.15252/embj.2018100926 (PMC6600649; doi:10.15252/embj.2018100926)
Supplement: Supplementary file 1 — Appendix [file EMBJ-38-e100926-s001.pdf]

Appendix for:

Human GBP1 is a microbe-specific gatekeeper of macrophage apoptosis and pyroptosis

Daniel Fisch[1,2], Hironori Bando[3,4], Barbara Clough[1], Veit Hornung[5], Masahiro Yamamoto[3,4], Avinash Shenoy[2,6], Eva-Maria Frickel[1,7*]

[1] Host-*Toxoplasma* Interaction Laboratory, The Francis Crick Institute, London, UK

[2] MRC Centre for Molecular Bacteriology & Infection, Imperial College, London, UK

[3] Department of Immunoparasitology, Research Institute for Microbial Diseases, Osaka University, Osaka, Japan

[4] Laboratory of Immunoparasitology, WPI Immunology Frontier Research Center, Osaka University, Osaka, Japan

[5] Gene Center and Department of Biochemistry & Center for Integrated Protein Science (CIPSM), Ludwig-Maximilians-Universität München, Munich, Germany

[6] The Francis Crick Institute, London, UK

*To whom correspondence should be addressed:

Avinash Shenoy, a.shenoy@imperial.ac.uk

Eva-Maria Frickel, eva.frickel@crick.ac.uk

[7] Lead contact: Eva-Maria Frickel, eva.frickel@crick.ac.uk

Table of contents:

1. Appendix Figure S1: Efficiency of siRNA-mediated silencing of expression of genes and protein levels in THP-1 and primary monocyte derived macrophages

2. Appendix Figure S2: Anti-GBP1 antibody validation for immunofluorescence stainings

3. Appendix Table S1: Cell lines

3. Appendix Table S2: Antibodies

4. Appendix Table S3: Primers for qRT-PCR

5. Appendix Table S4: Primers for cloning

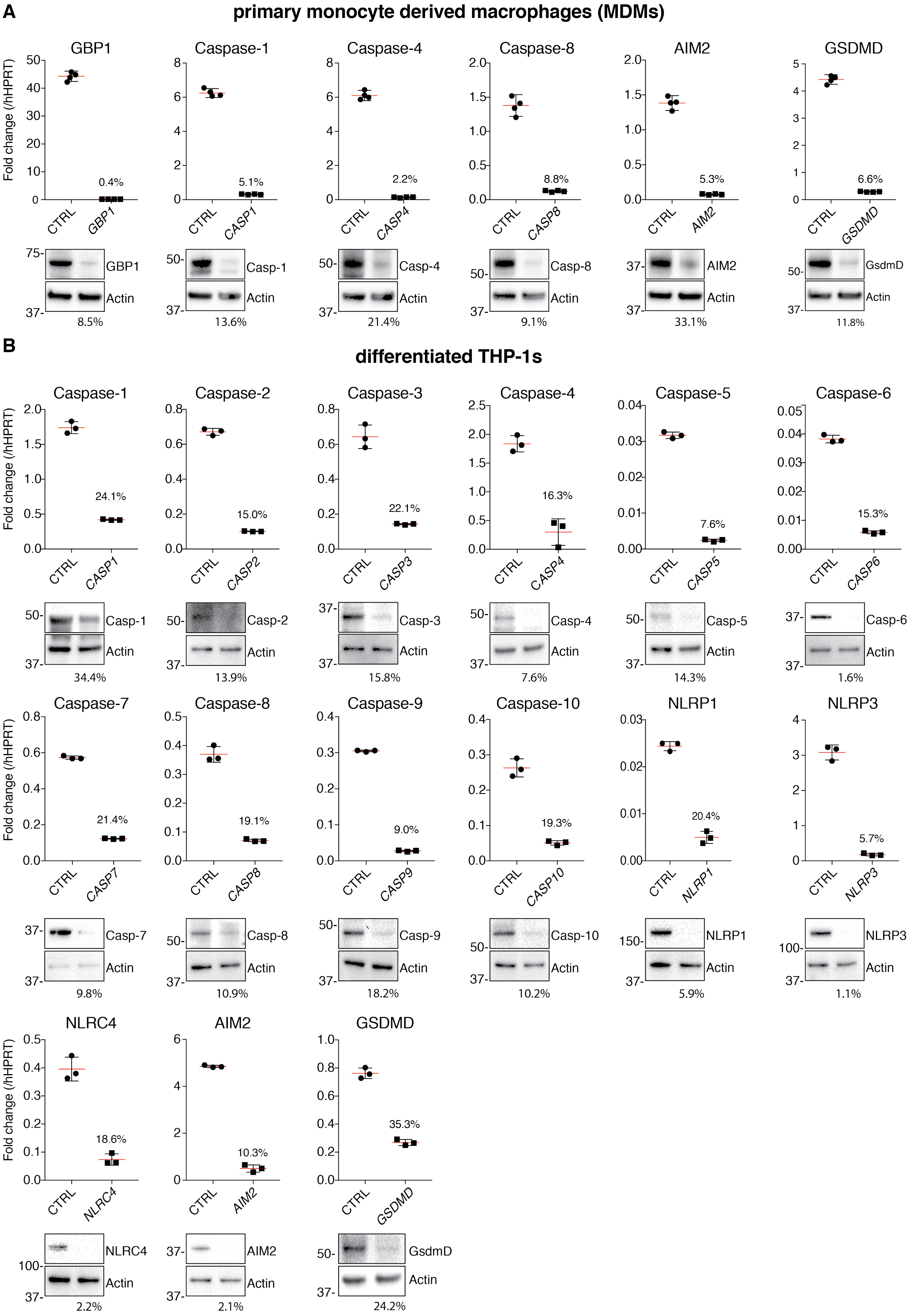
A
primary monocyte derived macrophages (MDMs)
GBP1
Caspase-1
Caspase-4
Caspase-8
AIM2
GSDMD
Fold change (/hHPRT)
CTRL
GBP1
CASP1
CASP4
CASP8
AIM2
GSDMD
0.4%
5.1%
2.2%
8.8%
5.3%
6.6%
Casp-1
Casp-4
Casp-8
GsdmD
Actin
8.5%
13.6%
21.4%
9.1%
33.1%
11.8%
B
differentiated THP-1s
Caspase-2
Caspase-3
Caspase-5
Caspase-6
CASP2
CASP3
CASP5
CASP6
24.1%
15.0%
22.1%
16.3%
7.6%
15.3%
Casp-2
Casp-3
Casp-5
Casp-6
34.4%
13.9%
15.8%
7.6%
14.3%
1.6%
Caspase-7
Caspase-9
Caspase-10
NLRP1
NLRP3
CASP7
CASP9
CASP10
21.4%
19.1%
9.0%
19.3%
20.4%
5.7%
Casp-7
Casp-9
Casp-10
9.8%
10.9%
18.2%
10.2%
5.9%
1.1%
NLRC4
18.6%
10.3%
35.3%
2.2%
2.1%
24.2%

Appendix Figure S1: Efficiency of siRNA-mediated silencing of expression of genes and protein levels in THP-1 and primary monocyte derived macrophages

(A-B) Graphs showing mRNA levels (normalized to *HPRT1*) and representative immunoblots from primary, IFNγ-primed MDMs **(A)** or PMA-differentiated and IFNγ-primed THP-1 **(B)**. Percentage mRNA levels or protein intensity as compared to cells transfected with nontargeting control siRNA (CTRL) are indicated. Mean ± SEM from n = 3 independent experiments (THP-1) or n = 4 independent experiments (MDMs) plotted.

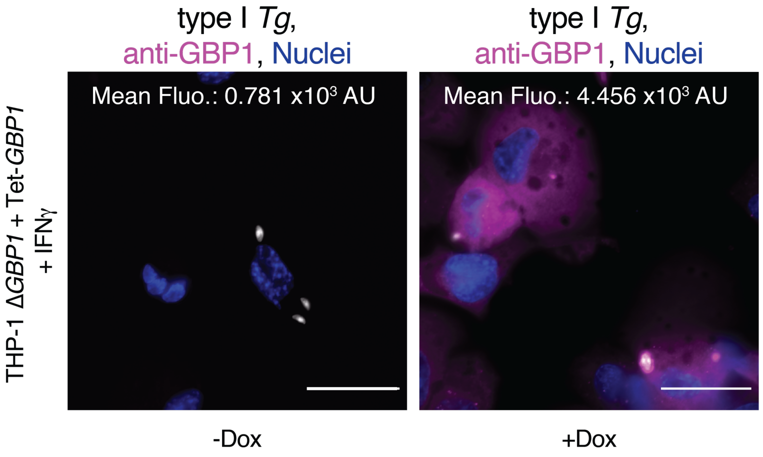

Appendix Figure S2: Anti-GBP1 antibody validation for immunofluorescence stainings

Immunofluorescence staining of IFNγ-primed THP-1 Δ*GBP1*+Tet-*GBP1* cells (all GBPs present but GBP1) infected with type I *Tg* (grey) at 6 hours post infection. Cells were left untreated or pre-treated with Doxycycline (Dox) to induce GBP1 expression and stained with home-made anti-GBP1 antibody (magenta). Mean GBP1 fluorescence signal (Mean Fluo.) is indicated in the images. Scale bar, 40 μm.

Appendix Table S1: Cell lines

**Cell line**	**Description**
A549	Adenocarcinoma alveolar basal epithelial cells
HEK 293T	Human embryonic kidney cells for virus production
HFF	Human foreskin fibroblasts for culturing *Toxoplasma gondii*
MDMs	Monocyte derived macrophages
THP-1 Δ*ASC*	THP-1, knockout for *ASC*
THP-1 Δ*CASP1*	THP-1, knockout for *CASP1*
THP-1 Δ*CASP4*	THP-1, knockout for *CASP4*
THP-1 Δ*GBP1*	THP-1, knockout for *GBP1*
THP-1 Δ*GBP1*+Tet	THP-1, knockout for *GBP1*, expressing Tet transactivator proteins
THP-1 Δ*GBP1*+Tet-*GBP1*	THP-1, knockout for *GBP1*, expressing Doxycycline-inducible GBP1 WT
THP-1 Δ*GBP1*+Tet-*GBP1* Δ589-592	THP-1, knockout for *GBP1*, expressing Doxycycline-inducible GBP1 Δ589-592 = CaaX box deletion
THP-1 Δ*GBP1*+Tet-*GBP1* C589A	THP-1, knockout for *GBP1*, expressing Doxycycline-inducible GBP1 C589A = no lipidation of CaaX box
THP-1 Δ*GBP1*+Tet-*GBP1* K382R	THP-1, knockout for *GBP1*, expressing Doxycycline-inducible GBP1 K382R = mutated ubiquitination site
THP-1 Δ*GBP1*+Tet-*GBP1* K51A	THP-1, knockout for *GBP1*, expressing Doxycycline-inducible GBP1 K51A = GTPase dead
THP-1 Δ*GBP1*+Tet-*GBP1* RK227/228EE	THP-1, knockout for *GBP1*, expressing Doxycycline-inducible GBP1 RK227/228EE = constitutively dimeric
THP-1 Δ*GBP1*+Tet-mCH-*GBP1*	THP-1, knockout for *GBP1*, expressing Doxycycline-inducible mCherry-tagged GBP1 WT
THP-1 Δ*GBP1* + Tet-mCH-*GBP1* Δ589-592	THP-1, knockout for *GBP1*, expressing Doxycycline-inducible mCherry-tagged GBP1 Δ589-592 = CaaX box deletion
THP-1 Δ*GBP1*+Tet-mCH-*GBP1* + YFP-*CASP4*	THP-1, knockout for *GBP1*, expressing Doxycycline-inducible mCherry-tagged GBP1 WT and YFP-tagged caspase-4

THP-1 ∆GBP1+Tet-mCH-*GBP1* + YFP-*CASP4* C258S	THP-1, knockout for *GBP1*, expressing Doxycycline-inducible mCherry-tagged GBP1 WT and YFP-tagged caspase-4 C258S = catalytically inactive
THP-1 ∆*GBP1*+Tet-mCH-*GBP1* C589A	THP-1, knockout for *GBP1*, expressing Doxycycline-inducible mCherry-tagged GBP1 C589A = no lipidation of CaaX box
THP-1 ∆*GBP1*+Tet-mCH-*GBP1* K51A	THP-1, knockout for *GBP1*, expressing Doxycycline-inducible mCherry-tagged GBP1 K51A = GTPase dead
THP-1 sh*GSDMD*	THP-1, stable knockdown of *GSDMD*
THP-1 WT	THP-1 monocytes, differentiated to macrophage-like cells with PMA

Appendix Table S2: Antibodies

**Target**	**Manufacturer**	**Cat number**
GBP1	homemade	-
Caspase-1	Adipogen	AG-20B-0048
Caspase-2	CST	#2224
Caspase-3	CST	#9661
Caspase-4	CST	#4450
Caspase-5	CST	#46680
Caspase-6	CST	#9762
Caspase-7	CST	#12827
Caspase-8	CST	#9746
Caspase-9	CST	#9508
Caspase-10	Genetex	GTX113148
Gasdermin D	CST	#96458
PARP	CST	#9542
NLRP1 (= NALP1)	CST	#4990
NLRP3	CST	#13158
NLRC4	CST	#12421
ASC	Adipogen	AG-25B-0006
AIM2	CST	#12948
Salmonella LPS	Abcam	ab8271
YFP	Abcam	ab6556

Appendix Table S3: Primers for qRT-PCR

Gene		Sequence 5'-3'
GBP1	fwd	TATTGCCCACTATGAACAGCAGAT
	rev	TAGCTGGGCCGCTAACTCC
GBP2	fwd	AATTAGGGGCCCAGTTGGAAG
	rev	AAGAGACGGTAACCTCCTGGT
GBP3	fwd	GAATAAGGGCTTCTCTCTGGGC
	rev	AGTGTCAAGCAGGACTAAGGTG
GBP4	fwd	TAAGCGGCTTTCAGAGCACC
	rev	GACCTCGTTTGCCTTAACTCC
GBP5	fwd	CCTGATGATGAGCTAGAGCCTG
	rev	GCACCAGGTTCTTTAGACGAGA
GBP6	fwd	TGCACCATCCCATTTGTGGAA
	rev	TGCCAACCTAGAAGAGCCTGC
GBP7	fwd	GAGTTAAGGCAGACGAGGTCC
	rev	TTCAGCTGCCTCCTTCTTAGC
ASC	fwd	ATCCAGGCCCCTCCTCAG
	rev	AGAGCTTCCGCATCTTGCTT
AIM2	fwd	ACAGGCCTGGATAACATCACT
	rev	ACCGCCCCAGCATTTTGAAT
NLRP1	fwd	GGACCAGTATCGAGAGCAGC
	rev	GAGGTGAGGATGGGTCTCCT
NLRP3	fwd	TCCTGGCTGTAACATTCGGAG
	rev	TGCAAGATCCTGACAACATGC

NLRC4	fwd	CGGCAGAGGGTTCTTTTCCT
	rev	ATATCCCCCACCTCAGCAGT
GsdmD	fwd	TAGTCCGGAGAGTGGTCCAG
	rev	ACCATGAGCTTGAGGGCTTC
Casp1	fwd	AATGGACAAGTCAAGCCGCA
	rev	AGTCATGTCCGAAGCAGTGAG
Casp2	fwd	GTTACCTGCACACCGAGTCA
	rev	TCAGTCTCATCTCCACGGCA
Casp3	fwd	CTCGGTCTGGTACAGATGTCGA
	rev	CATGGCTCAGAAGCACACAAAC
Casp4	fwd	CTGTTCCCTATGGCAGAAGGC
	rev	TCTGCCATGACCCGAACTTT
Casp5	fwd	GCAAGGAATGGGGCTCACTA
	rev	CGTGCTGTCAGAGGACTTGT
Casp6	fwd	AAATGGACCACAGGAGGAGAG
	rev	CTGAAAACCTGCGGGTAAGA
Casp7	fwd	ACATGAATTTTGAAAAGCTGGG
	rev	CAGGCGGCATTTGTATGGTC
Casp8	fwd	GGTCACTTGAACCTTGGGAA
	rev	AGGCCAGATCTTCACTGTC
Casp9	fwd	GTGGACATTGGTTCTGGAGGAT
	rev	CGCAACTTCTCACAGTCGATG
Casp10	fwd	ATCCTTTCGGCATGTGGAGG
	rev	TAGTGTGAAAGCAGGCTGGG

HPRT	fwd	ACCAGTCAACAGGGGACATAA
	rev	CTTCGTGGGGTCCTTTTCACC

Appendix Table S4: Primers for cloning

Name	Sequence 5'-3'	Purpose
LSC-5_repair_fwd	CGGGAGTATCCG	Deleting sgRNA cassette from pLSC-5 + Sequencing
LSC-5_repair_rev	AATTCGGATACTCCCGGTAC	
LSC-5_repair_seq	GGGACAGCAGAGATCCAGTT	
CMV_fwd	CGCAAATGGGCGGTAGGCGTG	Sequencing Tet vectors
EFS_fwd	TGAACGTTCTTTTTCGCAAC	
EFS_Seq_reverse	GTTGCGAAAAAGAACGTTCA	
mCH_seq-rev	CGAAGTTCATCACGCGCTC	
Tet-vector_Seq-fwd	TCCACGCTGTTTTGACCTC	
Tet_hGBP1_Seq_rev1	CTTTAGTGTGAGACTGCACCGTGG	
Tet-Off_BB-fwd	GACTTGAGCTGTCGCCGCCCCAGGCCATAAAGCGGCCGCGACTCTAGA	Cloning Tet-ctrl vector by Gibson assembly
Tet-ON_BB-rev	TATGACTTTGCTCTTGTCCAGTCTAGACATCCGTCGACTGCAGAATTCGAAGC	
BB_Tet-ON-fwd	AGCTCAAGCTTCGAATTCTGCAGTCGACGGATGTCTAGACTGGACAAGAGC	
P2A_Tet-ON-rev	CAGCAGAGAGAAGTTTGTTGCGCCGGATCCCCCGGGAGCATGT	

Tet-ON_P2A-fwd	GACGATTTTGACCTTGACATGCTCCCCGGGGGATCCGGCGCAACAAAC	
Tet-OFF_P2A-rev	GTTAATCACTTTACTTTTATCTAATCTAGACGGTCCAGGATTCTCTTCGAC	
P2A_Tet-OFF-fwd	GCCGGAGATGTCGAAGAGAATCCTGGACCGTCTAGATTAGATAAAAGTAAAGTGATTAACAGCG	
BB_Tet-OFF-rev	GGCTGATTATGATCTAGAGTCGCGGCCGCTTTATGGCCTGGGGCG	
bsd_BB-fwd	CTGCCCTCTGGTTATGTGTGGGAGGGCTAAAGCGGCCGCGACTCTAGA	
T2A_BB_rev	CAGCAGAGAGAAGTTTGTTGCGCCGGATCCTGGCCTGGGGCGGC	
BB_T2A_fwd	TTGGACTTGAGCTGTCGCCGCCCCAGGCCAGGATCCGGCGCAACAAAC	
bsd_T2A_rev	AATGAGGGTGGATTCTTCTTGAGACAAAGGCGGTCCAGGATTCTCTTCGAC	
T2A_bsd_fwd	GCCGGAGATGTCGAAGAGAATCCTGGACCGCCTTTGTCTCAAGAAGAATCCACC	
BB_bsd_rev	GGCTGATTATGATCTAGAGTCGCGGCCGCTTTAGCCCTCCCACACATAACC	
bsd_Seq_fwd	GATCGGAAATGAGAACAGGG	
BB_insert-fwd	TTGCCGCCAGAACACAGGACCGGTGCCACCATGTCTAGACTGGACAAGAGC	
BB_insert-rev	ATCCAGAGGTTGATTGTCGACTTAACGCGTTTAGCCCTCCCACACATAAC	
Insert_BB-fwd	CTGCCCTCTGGTTATGTGTGGGAGGGCTAAACGCGTTAAGTCGACAATCAACCTC	

Insert_BB-rev	TATGACTTTGCTCTTGTCCAGTCTAGACATGGTGGCACCGGTCCTG	
BB_Zeo-fwd	TTGCCGCCAGAACACAGGACCGGTGCCACCATGGCCAAGTTGACCAGTG	Cloning pLenti-Tet vector
BB_Zeo-rev	ATCCAGAGGTTGATTGTCGACTTAACGCGTTCAGTCCTGCTCCTCGG	
Zeo_BB-fwd	GTGCACTTCGTGGCCGAGGAGCAGGACTGAACGCGTTAAGTCGACAATCAACCTC	
Zeo_BB-rev	CACCGGAACGGCACTGGTCAACTTGGCCATGGTGGCACCGGTCCTG	
BB_Tet-Prom-fwd	TCAAAATTTTCGGGTTTATTACAGGGACAGCTCGAGTCCGGATCTCGAC	
BB_Tet-Prom-rev	GGTACCTTAATTAACCAAACTGGATCTCTGCTAGTTGTGGTTTGTCCAAACTCATC	
Tet-Prom_BB_fwd	CATTGATGAGTTTGGACAAACCACAACTAGCAGAGATCCAGTTTGGTTAATTAAGG	
Tet_Prom_BB-rev	GACGTGCCCGGGTCGAGATCCGGACTCGAGCTGTCCCTGTAATAAACCCG	
BB_Tet-Prom-fwd2	TTTGGTTAATTAAGGTACCGGGAGTATCCGCTCGAGTCCGGATCTCGAC	
BB_Tet-Prom-rev2	CCACTCCTTTCAAGACCTAGCTAGCGAATTCTAGTTGTGGTTTGTCCAAACTCATC	
MCS-fwd	GATCCCTGATCGATCGGCCG	
MCS-rev	AATTCGGCCGATCGATCAGG	
hGBP1_K51A_fwd	GCCTCTACCGCACAGGCGCATCCTACCTGATGAACA	Site directed

hGBP1_K51A_rev	TGTTCATCAGGTAGGATGCGCCTGTGCGGTAGAGGC	mutagenesis of GBP1
hGBP1_RK227-228EE_fwd	GCATTTTTTCTTTGGGAAGAATTCCTCGATACAGAGTCTGGGCAGGTTA	
hGBP1_RK227-228EE_rev	TAACCTGCCCAGACTCTGTATCGAGGAATTCTTCCCAAAGAAAAAATGC	
hGBP1_K382R_fwd	GCCGCTAACTCCCTTTGAAATAGATGGTCCACATCT	
hGBP1_K382R_rev	AGATGTGGACCATCTATTTCAAAGGGAGTTAGCGGC	
BB_hGBP1_fwd	AATTAGCGCTACCGGTGCGGCCGCACCATGGCATCAGAGATCCACATGACAGG	Gibson assembly of GBP1 into pLenti-Tet and deleting the C-terminus/mutating C589
BB_hGBP1-wt_rev	CTAGCGAATTCGGCCGATCGATCAGGGATCTTAGCTTATGGTACATGCCTTTCGTCGTC	
BB_hGBP1-delta589-592_rev	CTAGCGAATTCGGCCGATCGATCAGGGATCTTATGCCTTTCGTCGTCTCATTTTCGTC	
BB_hGBP1-C589A_rev	CTAGCGAATTCGGCCGATCGATCAGGGATCTTAGCTTATGGTAGCTGCCTTTCGTCGTC	
hGBP1_mCH_rev	TGGGCCTGTCATGTGGATCTCTGATGCCATCTACTTGTACAGCTCGTCCATGCC	Adding mCH to the GBP1 N-terminus during Gibson assembly
mCH_hGBP1_fwd	TCCACCGGCGGCATGGACGAGCTGTACAAGATGGCATCAGAGATCCACATGACAGG	
Tet_mCH_Gibson_fwd	AATTAGCGCTACCGGTGCGGCCGCACCATGGTGAGCAAGGGCGAGG	

Tet_hGBP1_Seq_fwd1	CCACGGTGCAGTCTCACACTAAAG	Sequencing Tet-GBP1 vectors
Tet_hGBP1_Seq_fwd2	TCAATGCCATCAGCAGTGGG	
Tet_hGBP1_Seq_fwd3	GAAGCATCATCAGATCGTTGC	
ACTB-ctrl_fwd	GTGCTATCCCTGTACGCCTC	PCR ctrl for genotyping
ACTB-ctrl_rev	GCAGCTCGTAGCTCTTCTCC	
GBP1_cDNA_fwd	GCCTGGACATGGCATCAGAG	Amplifying GBP ORFs for sequencing and subcloning
GBP1_cDNA_rev	GGTGACAGGAAGGCTCTGG	
GBP2_cDNA_fwd	CCTGGACATGGCTCCAGAGA	
GBP2_cDNA_rev	GCTGGACAGGCAAATTTTGCTC	
GBP3_cDNA_fwd	CAGACAAGAGAACAATGCCCTGG	
GBP3_cDNA_rev	GCTCTGTTGTTTAGATCTTTAGCTTATGCG	
GBP4_cDNA_fwd	GAGGACAGAGCAATGGGTGAG	
GBP4_cDNA_rev	CAGGCTCTTAAATACGTGAGCCAAG	
GBP5_cDNA_fwd	CATCCTAGACATGGCTTTAGAGATCCAC	

GBP5_cDNA_rev	CTCCCATATTTAGCACTTTAGAGTAAAACACATG	
GBP1_gRNA1_fwd	CACCGAGGCTTCTCTCTGGGCTCCA	Cloning GBP1 gRNA1 plasmid
GBP1_gRNA1_rev	AAACTGGAGCCCAGAGAGAAGCCTC	
GBP1_gRNA2_fwd	CACCGGGCTTCAGCAAAAATGTTGC	Cloning GBP1 gRNA2 plasmid
GBP1_gRNA2_rev	AAACGCAACATTTTTGCTGAAGCCC	
GBP1-KO check1_fwd	CATGGCATTTATTATGTTGAGGTGC	gDNA Sequence analysis for CRISPR result
GBP1-KO check1_rev	ATTTGTGCTCCCTTTATGCATCTGT	
GBP1-KO check2_fwd	GATTGAAGGTGAGGAGTGAGTTAAG	
GBP1-KO check2_rev	AGTACAAGTAAGCAAGCAGGGTCCT	
CASP4-EcoRI-fwd	atctcgagctcaagcttcgATGGCAGAAGGCAACCACA	Cloning pMX-YFP-Caspase-4
CASP4-EcoRI-rvs	ctcgaggcctgcaggaattcTCAATTGCCAGGAAAGAGGTA	
CASP4-C258S-fwd	catcattgtccaggccTCCagaggtgcaaacc	Site-directed mutagenesis
CASP4-C258S-rvs	ggtttgcacctctGGAggcctggacaatgatg	
