# Supplementary material for: Human GBP1 is a microbe‐specific gatekeeper of macrophage apoptosis and pyroptosis
Source: EMBO J. 2019 Jun 3;38(13):e100926. doi: 10.15252/embj.2018100926 (PMC6600649; doi:10.15252/embj.2018100926)
Supplement: Supplementary file 2 — Expanded View Figures PDF [file EMBJ-38-e100926-s002.pdf]

Expanded View Figures

Figure EV1. Quality control of GBP ablation in THP-1 cells.

A Fold-change of mRNA of *GBP1-7* compared to hypoxanthine phosphoribosyltransferase 1 (*HPRT1*) in PMA-differentiated THP-1 cells (left) and primary MDMs (right) following treatment with IFNγ (50 IU/ml). Mean (red bar) ± SEM of n = 14 experiments (THP-1) or n = 6 experiment (MDMs) shown; n.d. not detected.

B Immunoblots from differentiated THP-1 WT or MDMs treated with IFNγ (50 IU/ml) or left untreated. Images represent n = 3 independent experiments.

C qRT–PCR measurement of silencing of expression of the indicated *GBPs* in IFNγ-primed THP-1 cells transfected with siRNA against *GBP1-5*. mRNA fold-change (mean ± SEM; n = 3 independent experiments) normalized to *HPRT1* as percentage of cells transfected with non-targeting control (CTRL) siRNA is indicated.

D Immunoblots from indicated THP-1 cells treated with IFNγ. Images represent n = 3 independent experiments.

E qRT–PCR measurement of *GBP1-5* expression in THP-1 and THP-1 Δ*GBP1* cells treated with IFNγ plotted as fold-change to *HPRT1*. Mean ± SEM plotted from n = 3 experiments; n.d. not detected.

F RT–PCR amplification of *GBP1-5* coding sequences (CDS) from indicated THP-1 cells treated with IFNγ. Yellow arrowhead indicates the truncated GBP1 CDS in the Δ*GBP1* cells.

G Sequencing results showing loss of *GBP1* coding region. Top: Needleman-Wunsch alignment of *GBP1* sequence from THP-1 Δ*GBP1* and *GBP1* transcript sequence NM_002053.2 showing the deletion in knockout cells. Bottom left: *GBP1* CDS with deletion highlighted in red. qRT–PCR primer binding sites marked in blue and bold letters and amplicon marked in blue. Bottom right: Summary table of sequencing result of *GBP1-5* in THP-1 Δ*GBP1* cells confirming loss of *GBP1* and wild-type coding sequences of *GBP2-5*.

© 2019 The Authors

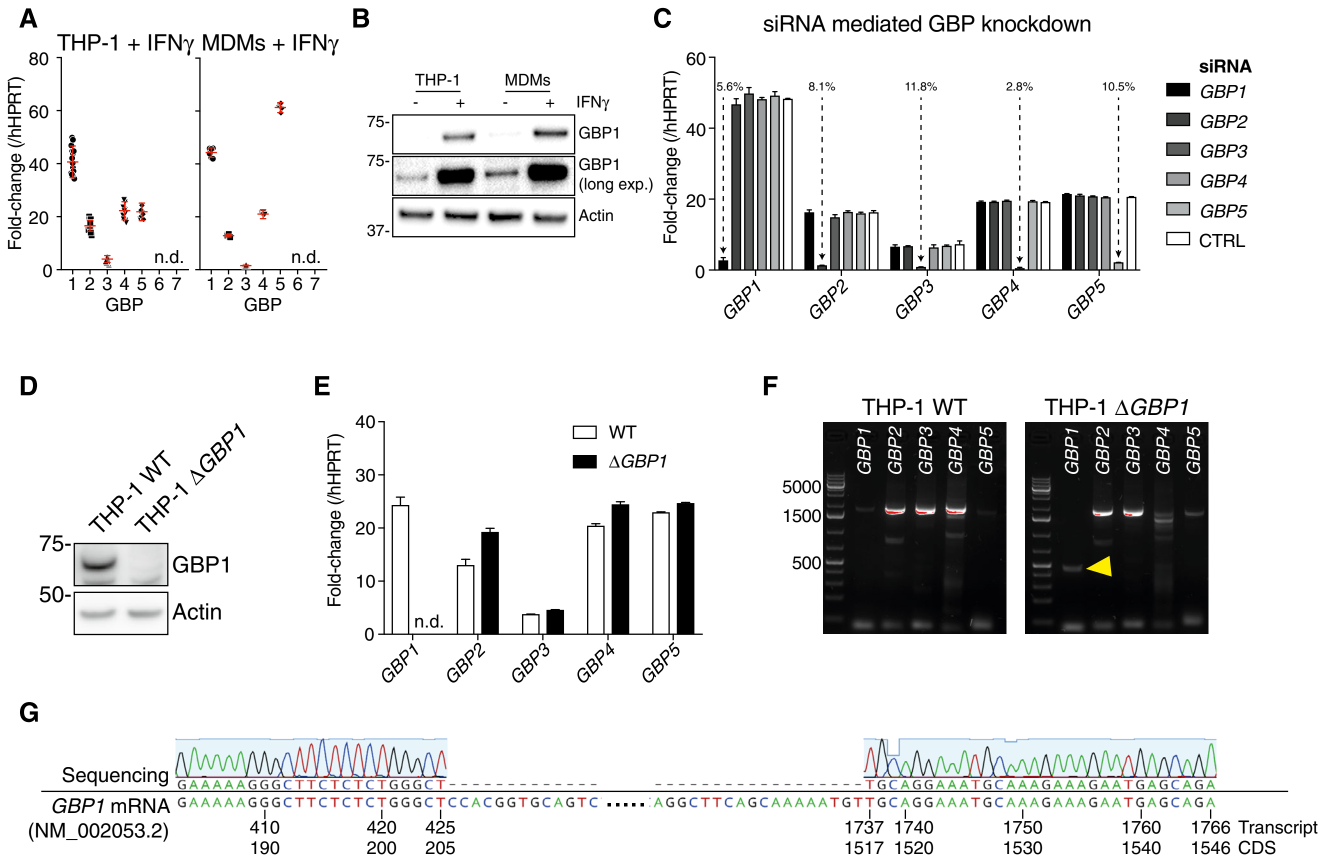

GBP1 coding sequence of the mRNA (NM_002053.2):

```
ATGGCATCAGAGATCCACATGACAGGCCCAATGTGCCTCATTGAGAACACTAA
TGGGCGACTGATGGCGAATCCAGAAGCTCTGAAGATCCTTTCTGCCATTACAC
AGCCTATGGTGGTGGTGGCAATTGTGGGCCTCTACCGCACAGGCAAATCCTAC
CTGATGAACAAGCTGGCTGGAAAGAAAAAGGGCTTCTCTCTGGGCTCCACGGT
GCAGTCTCACACTAAAGGAATCTGGATGTGGTGTGTGCCCCACCCCAAGAAGC
CAGGCCACATCCTAGTTCTGCTGGACACCGAGGGTCTGGGAGATGTAGAGAAG
GGTGACAACCAGAATGACTCCTGGATCTTCGCCCTGGCCGTCCTCCTGAGCAG
CACCTTCGTGTACAATAGCATAGGAACCATCAACCAGCAGGCTATGGACCAAC
TGTACTATGTGACAGAGCTGACACATAGAATCCGATCAAAATCCTCACCTGAT
GAGAATGAGAATGAGGTTGAGGATTCAGCTGACTTTGTGAGCTTCTTCCCAGA
CTTTGTGTGGACACTGAGAGATTTCTCCCTGGACTTGGAAGCAGATGGACAAC
CCCTCACACCAGATGAGTACCTGACATACTCCCTGAAGCTGAAGAAAGGTACC
AGTCAAAAAGATGAAACTTTTAACCTGCCCAGACTCTGTATCCGGAAATTCTT
CCCAAAGAAAAAATGCTTTGTCTTTGATCGGCCCGTTCACCGCAGGAAGCTTG
CCCAGCTCGAGAAACTACAAGATGAAGAGCTGGACCCCGAATTTGTGCAACAA
GTAGCAGACTTCTGTTCCTACATCTTTAGTAATTCCAAAACTAAAACTCTTTC
AGGAGGCATCCAGGTCAACGGGCCTCGTCTAGAGAGCCTGGTGCTGACCTACG
TCAATGCCATCAGCAGTGGGGATCTGCCGTGCATGGAGAACGCAGTCCTGGCC
TTGGCCCAGATAGAGAACTCAGCTGCAGTGCAAAAGGCTATTGCCCACTATGA
ACAGCAGATGGGCCAGAAGGTGCAGCTGCCCACAGAAACCCTCCAGGAGCTGC
TGGACCTGCACAGGGACAGTGAGAGAGAGGCCATTGAAGTCTTCATCAGGAGT
TCCTTCAAAGATGTGGACCATCTATTTCAAAAGGAGTTAGCGGCCCAGCTAGA
AAAAAAGCGGGATGACTTTTGTAAACAGAATCAGGAAGCATCATCAGATCGTT
GCTCAGCTTTACTTCAGGTCATTTTCAGTCCTCTAGAAGAAGAAGTGAAGGCG
GGAATTTATTCGAAACCAGGGGGCTATCGTCTCTTTGTTCAGAAGCTACAAGA
CCTGAAGAAAAAGTACTATGAGGAACCGAGGAAGGGGATACAGGCTGAAGAGA
TTCTGCAGACATACTTGAAATCCAAGGAGTCTATGACTGATGCAATTCTCCAG
ACAGACCAGACTCTCACAGAAAAAGAAAAGGAGATTGAAGTGGAACGTGTGAA
AGCTGAGTCTGCACAGGCTTCAGCAAAAATGTTGCAGGAAATGCAAAGAAAGA
ATGAGCAGATGATGGAACAGAAGGAGAGGAGTTATCAGGAACACTTGAAACAA
CTGACTGAGAAGATGGAGAACGACAGGGTCCAGTTGCTGAAAGAGCAAGAGAG
GACCCTCGCTCTTAAACTTCAGGAACAGGAGCAACTACTAAAAGAGGGATTTC
AAAAAGAAAGCAGAATAATGAAAAATGAGATACAGGATCTCCAGACGAAAATG
AGACGACGAAAGGCATGTACCATAAGCTAA
```

Gene	Sequencing result
GBP1	Deletion 426-1736 on transcript, deletion after G68 and frameshift
GBP2	WT
GBP3	WT
GBP4	WT
GBP5	WT

Figure EV1.

Figure EV2. Doxycycline-inducible re-expression of GBP1 in THP-1 Δ*GBP1*.

A Graphical overview of the Doxycycline-inducible system. Tet-activator rtTA2S-M2 (TA), silencer tTS (TS), and bsr (encoding Blasticidin S resistance) are constitutively expressed using the Tet-ctrl vector. In the absence of Doxycycline (Dox), TS binds to the response vector encoding GBP1 open reading frame under the control of heptameric tet-operon (TetO7) and blocks GBP1 expression. Upon treatment with Dox, TA loses DNA binding capacity and TA induces GBP1 expression.

B, C Immunoblots of GBP1 and β-actin from THP-1, THP-1 Δ*GBP1*, and THP-1 Δ*GBP1* stably transduced with Tet-*GBP1* plasmid for Dox-inducible GBP1 expression. Cells were treated with IFNγ and/or Dox as indicated in (B) or indicated concentrations of Dox in (C). Images represent n = 3 independent experiments.

D Immunoblots of GBP1 and β-actin and plot of GBP1 band intensity from THP-1 Δ*GBP1* + Tet-*GBP1* cells pre-treated with Dox (0.2 μg/ml) for 12 h, washed-off, and cycloheximide (CHX; 50 μg/ml) added for times as indicated. Band intensities (mean ± SEM) quantified using FIJI of n = 3 independent experiments are plotted, and red line shows a non-linear fit of the data. The predicted GBP1 half-life τ = 6.33 h.

E Graphical representation of GBP1 mutations and their biological effect mapped on the GBP1 primary structure (not to scale). NTHD = *N*-terminal helical domain, CaaX = CaaX motif for prenylation. Amino acid positions in human GPB1 are indicated.

F Immunoblots of GBP1 and β-actin from THP-1 wild-type or THP-1 Δ*GBP1* cells stably reconstituted with indicted Dox-inducible GBP1 variants or empty vector (EV). Cells were treated with either IFNγ or Dox.

 © 2019 The Authors

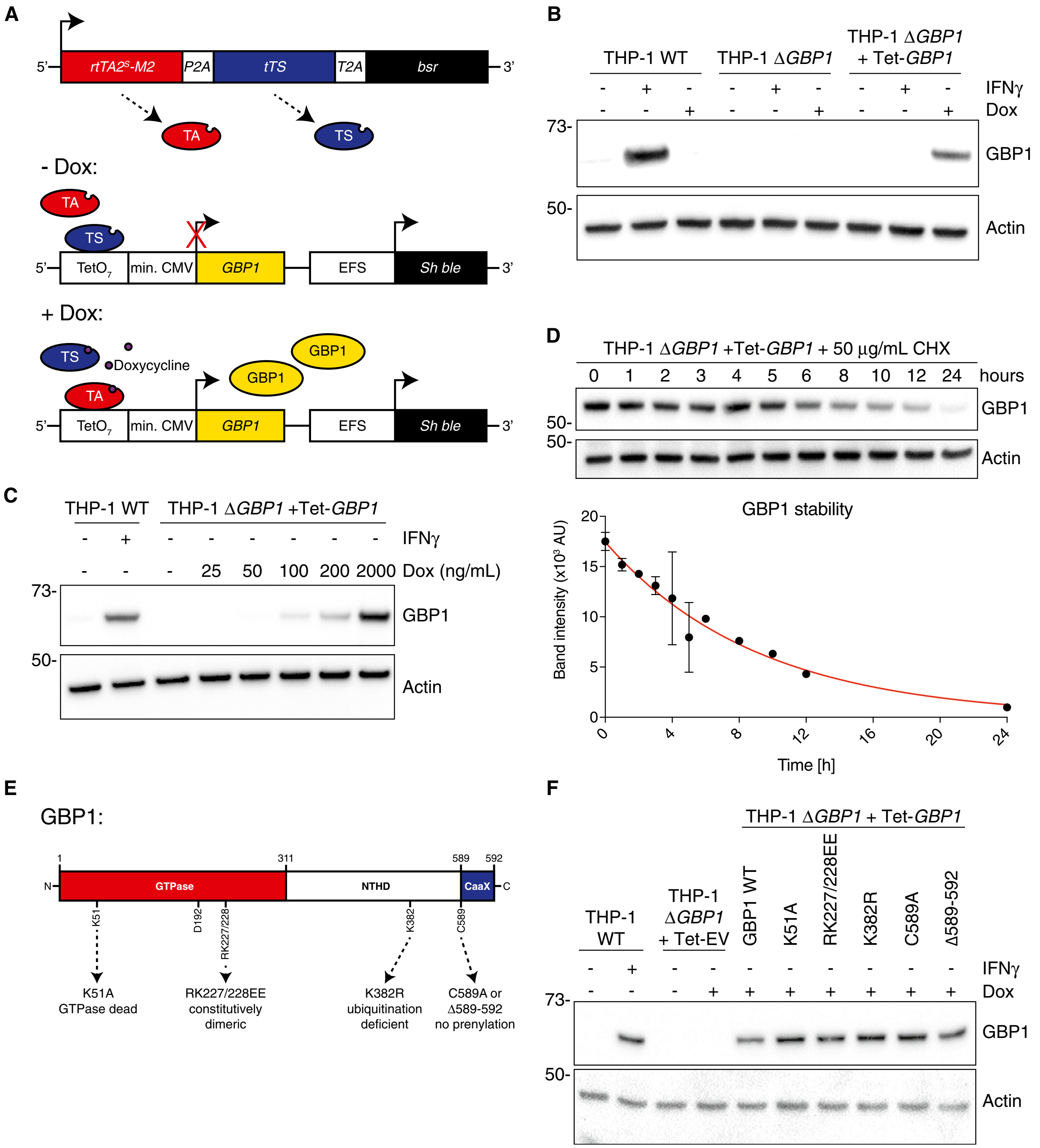
A
5' rtTA2S-M2 P2A tTS T2A bsr 3'
TA
TS
- Dox:
TetO7
min. CMV
GBP1
EFS
Sh ble
+ Dox:
Doxycycline
B
THP-1 WT
THP-1 ΔGBP1
THP-1 ΔGBP1 + Tet-GBP1
IFNγ
Dox
GBP1
Actin
73-
50-
C
THP-1 WT
THP-1 ΔGBP1 +Tet-GBP1
IFNγ
Dox (ng/mL)
25 50 100 200 2000
D
THP-1 ΔGBP1 +Tet-GBP1 + 50 µg/mL CHX
0 1 2 3 4 5 6 8 10 12 24 hours
GBP1 stability
Band intensity (x10³ AU)
Time [h]
E
GBP1:
GTPase
NTHD
CaaX
K51A GTPase dead
RK227/228EE constitutively dimeric
K382R ubiquitination deficient
C589A or Δ589-592 no prenylation
F
THP-1 ΔGBP1 + Tet-GBP1
THP-1 WT
THP-1 ΔGBP1 + Tet-EV
GBP1 WT
K51A
RK227/228EE
K382R
C589A
Δ589-592

Figure EV2.

Figure EV3. *Toxoplasma* infection induces apoptosis in human macrophages.

A Propidium iodide (PI) uptake assay of naïve or IFNγ-primed THP-1 cells infected with either type I or type II *Toxoplasma gondii* (*Tg*) for 18 h in the absence or presence of pan-caspase inhibitors zVAD-fmk (25 μM) or qVD (20 μM). Area under the curve (AUC) from real-time assay plotted as mean ± SEM from n = 3 independent experiments.

B Immunoblots of the indicated proteins from THP-1 WT or Δ*GBP1* cells infected with type I and type II *Tg* for 6 h or left uninfected (UI). Cells were unprimed or primed with IFNγ as indicated. Cells were treated with Nigericin (Nig., 10 μM for 2 h) to activate the NLRP3–caspase-1 pathway as a positive control. Images represent n = 3 independent experiments.

C ELISA for IL-1β (left) or IL-18 (middle) from IFNγ-primed THP-1 cells or IL-1β from primary MDMs infected with *Tg* for 24 h. Mean ± SEM are shown from n = 6 experiments (IL-1β ELISA THP-1), 3 experiments (IL-18 ELISA THP-1), and 4 donors of MDMs. Dashed lines for the +IFNγ data points indicate matching values from the same experiment.

D Real-time annexin V (AnnV)-Glo assay from indicated IFNγ-primed THP-1 cells left uninfected or infected with type I or type II *Tg* for 18 h. Area under the curve (AUC) from real-time assays plotted as mean ± SEM from n = 3 independent experiments.

E Quantification of apoptosis by the AnnV-staining assay in indicated THP-1 cells infected with *Tg*. Upper panels show representative flow cytometry scatter plots of unprimed or IFNγ-primed THP-1 cells uninfected or infected with GFP-expressing *Tg* strains as indicated. Cells were gated for single cells and GFP. Graph (below) shows the quantification (mean ± SEM from n = 3 independent experiments) of flow cytometry data of $AnnV^+$ cells.

F AnnV-Glo assay of THP-1 WT, Δ*GBP1*, and Δ*GBP1* cells stably reconstituted with Tet-wild-type GBP1 or $GBP1^{K51A}$, $GBP1^{C589A}$, or $GBP1^{\Delta 589–592}$ mutants as indicated, left untreated, or treated with Staurosporine (Sts) or TNFα + cycloheximide (TNFα + CHX) for 18 h. Area under the curve (AUC) from real-time assays plotted as mean ± SEM from n = 3 independent experiments.

G Immunoblot of IFNγ-primed THP-1 infected with type I or type II *Tg* for 12 h. As positive control, cells were treated with TNFα + CHX. Representative image of n = 2 experiments.

H Representative immunofluorescence images of IFNγ-primed THP-1 WT cells infected with type I or type II *Tg* (gray) at 12 h post-infection. Merged fluorescence and bright-field image (left) and fluorescence only (right) are shown. Yellow arrowheads, apoptotic nuclei; Dotted line, cell outline; Gray, differential interference contrast; Blue, nuclei; Green, *Tg*. Scale bar, 20 μm.

I Immunoblot of IFNγ-primed THP-1 infected with type I or type II *Tg* for the indicated time.

Data information: $^*P \leq 0.05$; $^{**}P \leq 0.01$, $^{****}P \leq 0.0001$ in (A, C) from two-way ANOVA following adjustment for multiple comparisons; ns, not significant.

 © 2019 The Authors

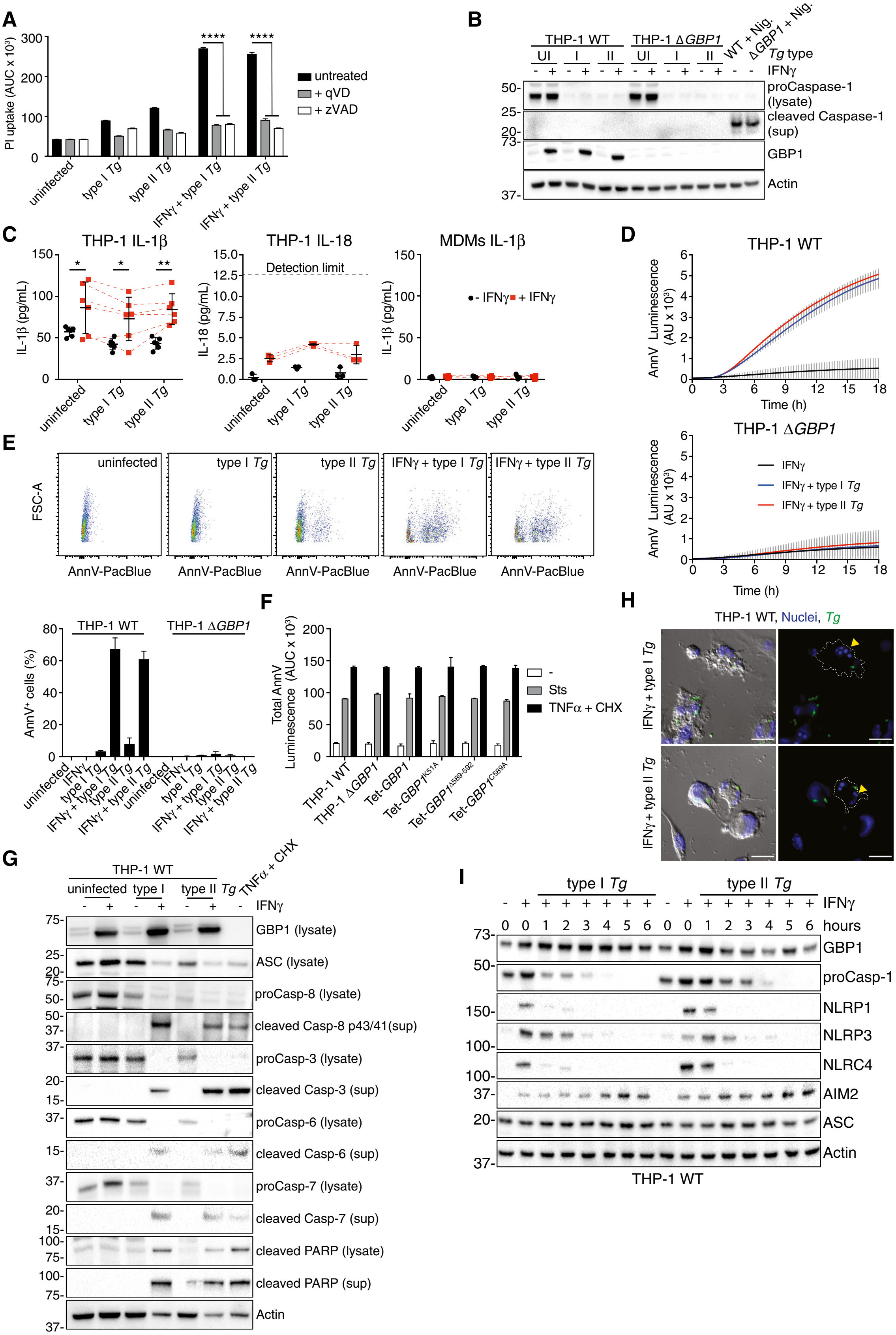
A
PI uptake (AUC x 10³)
untreated
+ qVD
+ zVAD
uninfected
type I Tg
type II Tg
IFNγ + type I Tg
IFNγ + type II Tg
B
THP-1 WT
THP-1 ΔGBP1
WT + Nig.
ΔGBP1 + Nig.
Tg type
IFNγ
proCaspase-1 (lysate)
cleaved Caspase-1 (sup)
GBP1
Actin
C
THP-1 IL-1β
THP-1 IL-18
MDMs IL-1β
IL-1β (pg/mL)
IL-18 (pg/mL)
Detection limit
- IFNγ + IFNγ
D
THP-1 WT
THP-1 ΔGBP1
AnnV Luminescence (AU x 10³)
Time (h)
IFNγ
IFNγ + type I Tg
IFNγ + type II Tg
E
FSC-A
AnnV-PacBlue
uninfected
type I Tg
type II Tg
IFNγ + type I Tg
IFNγ + type II Tg
AnnV⁺ cells (%)
THP-1 WT
THP-1 ΔGBP1
F
Total AnnV Luminescence (AUC x 10³)
-
Sts
TNFα + CHX
THP-1 WT
THP-1 ΔGBP1
Tet-GBP1
Tet-GBP1K51A
Tet-GBP1Δ589-592
Tet-GBP1C589A
H
THP-1 WT, Nuclei, Tg
IFNγ + type I Tg
IFNγ + type II Tg
G
THP-1 WT
uninfected
type I
type II Tg
TNFα + CHX
IFNγ
GBP1 (lysate)
ASC (lysate)
proCasp-8 (lysate)
cleaved Casp-8 p43/41(sup)
proCasp-3 (lysate)
cleaved Casp-3 (sup)
proCasp-6 (lysate)
cleaved Casp-6 (sup)
proCasp-7 (lysate)
cleaved Casp-7 (sup)
cleaved PARP (lysate)
cleaved PARP (sup)
Actin
I
type I Tg
type II Tg
IFNγ
hours
GBP1
proCasp-1
NLRP1
NLRP3
NLRC4
AIM2
ASC
Actin
THP-1 WT

Figure EV3.

Figure EV4. *Toxoplasma* infection blocks pyroptosis, but GBP1 enhances *Salmonella*-induced pyroptosis via caspase-4.

A AnnV-Glo assay of IFNγ-primed THP-1 WT, Δ*CASP1, or* Δ*CASP4* cells transfected with siCTRL or si*CASP8* and infected with either type I or type II *Toxoplasma gondii* (*Tg*) for 18 h. Area under the curve (AUC) from real-time assays plotted as mean ± SEM from *n* = 3 independent experiments.

B Propidium iodide (PI) uptake assay from naïve or IFNγ-primed THP-1 left uninfected or infected with type I or type II *Tg* for 4 h. Cells were treated with nigericin at 2 h post-infection to activate NLRP3–caspase-1. Area under the curve (AUC) from real-time assays plotted as mean ± SEM from *n* = 3 independent experiments.

C AnnV-Glo assay of THP-1 Δ*GBP1 + Tet-GBP1* cells left untreated or treated with IFNγ, Doxycycline (Dox), or both and infected with either type I or type II *Tg* for 18 h. Area under the curve (AUC) from real-time assays plotted as mean ± SEM from *n* = 3 independent experiments.

D LDH release assays from A549 epithelial cells primed with IFNγ or left untreated and infected type I or type II *Tg* for 24 h or uninfected (UI). As positive control, cells were treated with TNFα and cycloheximide (CHX). Mean ± SEM from *n* = 3 independent experiments.

E LDH release assay (left) or PI uptake assay (right) from IFNγ-primed or untreated THP-1 WT infected with *Salmonella* Typhimurium (STm) or STm-GFP (MOI = 30) for 4 h. Mean ± SEM of LDH assay or from area under the curve (AUC) of the PI uptake real-time assays plotted from *n* = 3 independent experiments.

F, G LDH release assays (F) or IL-1β ELISA (G) from THP-1 and primary MDMs primed with IFNγ and infected with STm-GFP (MOI = 30) at 4 h post-infection. Mean ± SEM from *n* = 3 (THP-1) or *n* = 4 (MDMs) independent experiments plotted.

H PI uptake assay of THP-1-, Δ*CASP1*-, Δ*CASP4*-, or *GSDMD*-silenced cells ($GSDMD^{miR}$) primed with IFNγ and infected with STm-GFP. Mean ± SEM from area under the curve (AUC) of 4 h real-time assays plotted from *n* = 3 independent experiments.

I PI uptake assay from IFNγ-primed THP-1 Δ*GBP1 + Tet-GBP1* cells transfected with non-targeting control (CTRL) or *CASP1*, *CASP4*, and *GSDMD* siRNA and infected with STm-GFP for 4 h. Mean ± SEM from area under the curve (AUC) from real-time assays plotted from *n* = 3 independent experiments.

J PI uptake assay of IFNγ-primed THP-1 or THP-1 Δ*GBP1* cells stably expressing indicated mutants of GBP1 (Δ*GBP1* + Tet-*GBP1*) treated with Dox and treated with Nigericin to induce NLRP3-dependent pyroptosis. Mean ± SEM from area under the curve (AUC) of 4-h real-time assays plotted from *n* = 3 independent experiments.

K Immunoblots of GBP1, YFP, and β-actin from THP-1 Δ*GBP1* + Tet-mCH-*GBP1* and THP-1 Δ*GBP1* + Tet-mCH-*GBP1* stably transduced with YFP-$CASP4^{C258S}$. Cells were treated with IFNγ and/or Dox as indicated. Images represent *n* = 3 independent experiments.

Data information: *$P \le 0.05$; **$P \le 0.01$, ***$P \le 0.001$, ****$P \le 0.0001$ from two-way ANOVA following adjustment for multiple comparisons; ns, not significant.

 © 2019 The Authors

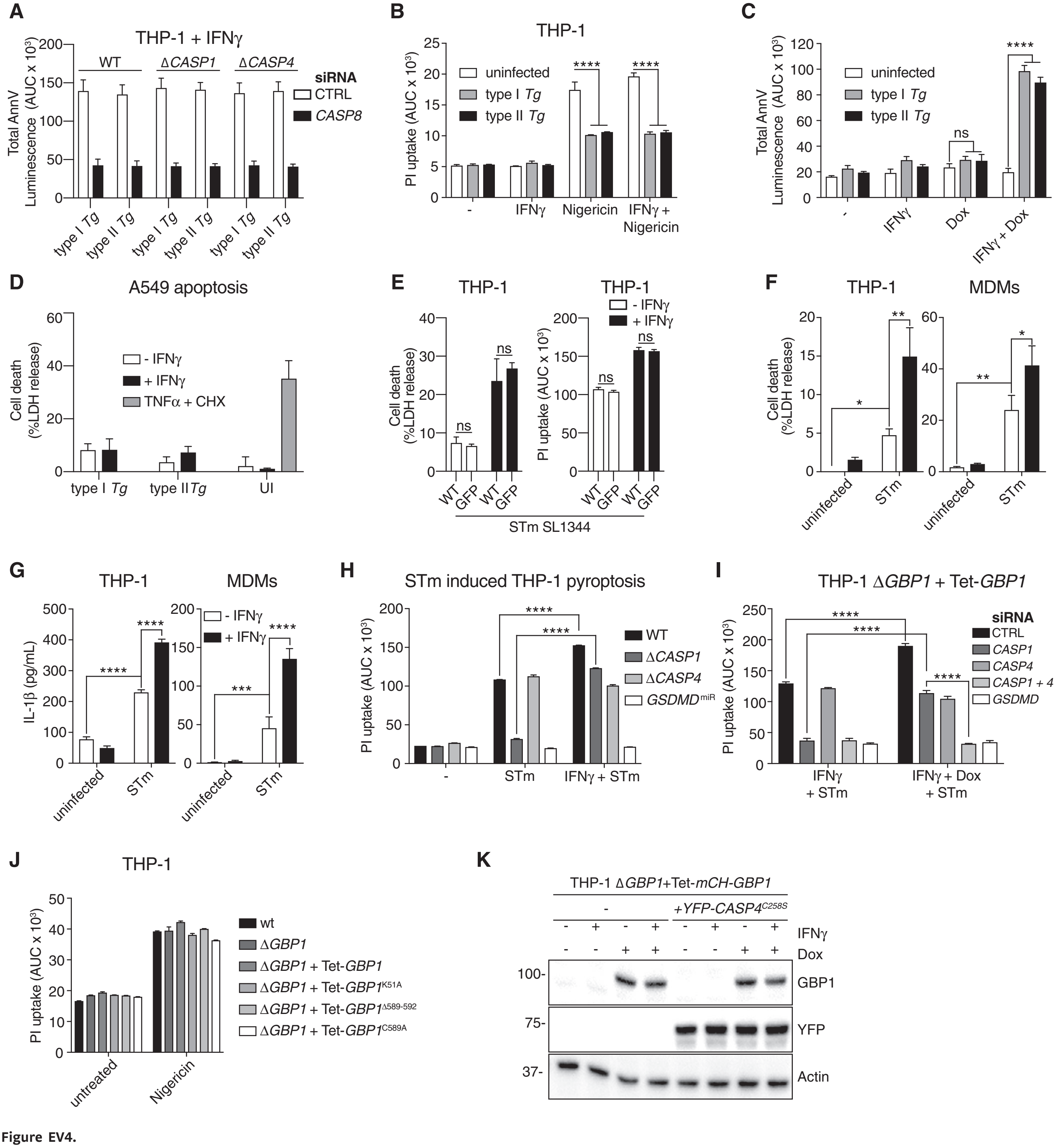
A
THP-1 + IFNγ
B
THP-1
C
D
A549 apoptosis
E
THP-1
THP-1
STm SL1344
F
THP-1
MDMs
G
THP-1
MDMs
H
STm induced THP-1 pyroptosis
I
THP-1 ΔGBP1 + Tet-GBP1
J
THP-1
K
THP-1 ΔGBP1+Tet-mCH-GBP1
+YFP-CASP4C258S
IFNγ
Dox
GBP1
YFP
Actin

Figure EV4.

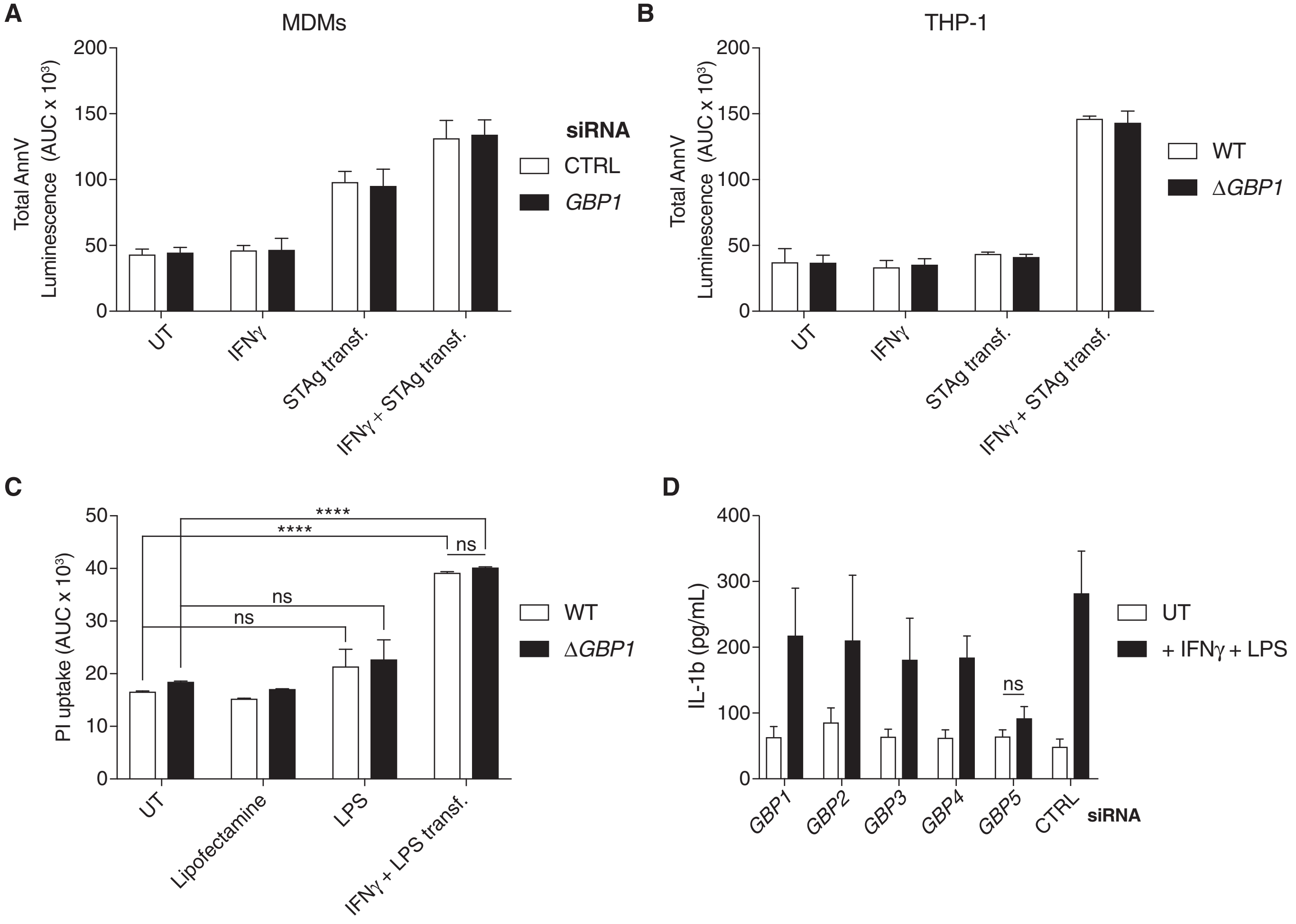

Figure EV5. Transfection of pathogen-derived ligands bypasses the requirement for GBP1 in cell death.

A, B Annexin V (AnnV)-Glo assay kinetic of primary monocyte-derived macrophages (MDM) transfected with non-targeting control (CTRL) or *GBP1* siRNA (A) and THP-1 or Δ*GBP1* cells (B) and transfected with soluble *Toxoplasma gondii* antigen (STAg). Cells were unprimed or primed with IFNγ before experiments. Mean ± SEM from area under the curve (AUC) of 18-h real-time assays plotted from $n = 4$ independent experiments.

C Propidium iodide (PI) uptake assay from indicated THP-1 cells left untreated (UT), treated with LPS or transfection reagent (Lipofectamine) only, or transfected with LPS (for 4 h) with IFNγ-priming. Area under the curve (AUC) from real-time assay plotted as mean ± SEM from $n = 3$ independent experiments.

D IL-1β ELISA from THP-1 cells transfected with non-targeting control (CTRL) or *GBP1-5* siRNA as indicated which were left untreated or primed with IFNγ and transfected with LPS. Mean ± SEM from area under the curve (AUC) of 24-h real-time assays plotted from $n = 3$ independent experiments.

Data information: ****$P \leq 0.0001$ from two-way ANOVA following adjustment for multiple comparisons; ns, not significant.

 © 2019 The Authors
