# Supplementary material for: Human GBP1 is a microbe‐specific gatekeeper of macrophage apoptosis and pyroptosis
Source: EMBO J. 2019 Jun 3;38(13):e100926. doi: 10.15252/embj.2018100926 (PMC6600649; doi:10.15252/embj.2018100926)
Supplement: Supplementary file 5 — Source Data for Figure 3 [file EMBJ-38-e100926-s004.pdf]

Immunoblots from Figure 3D

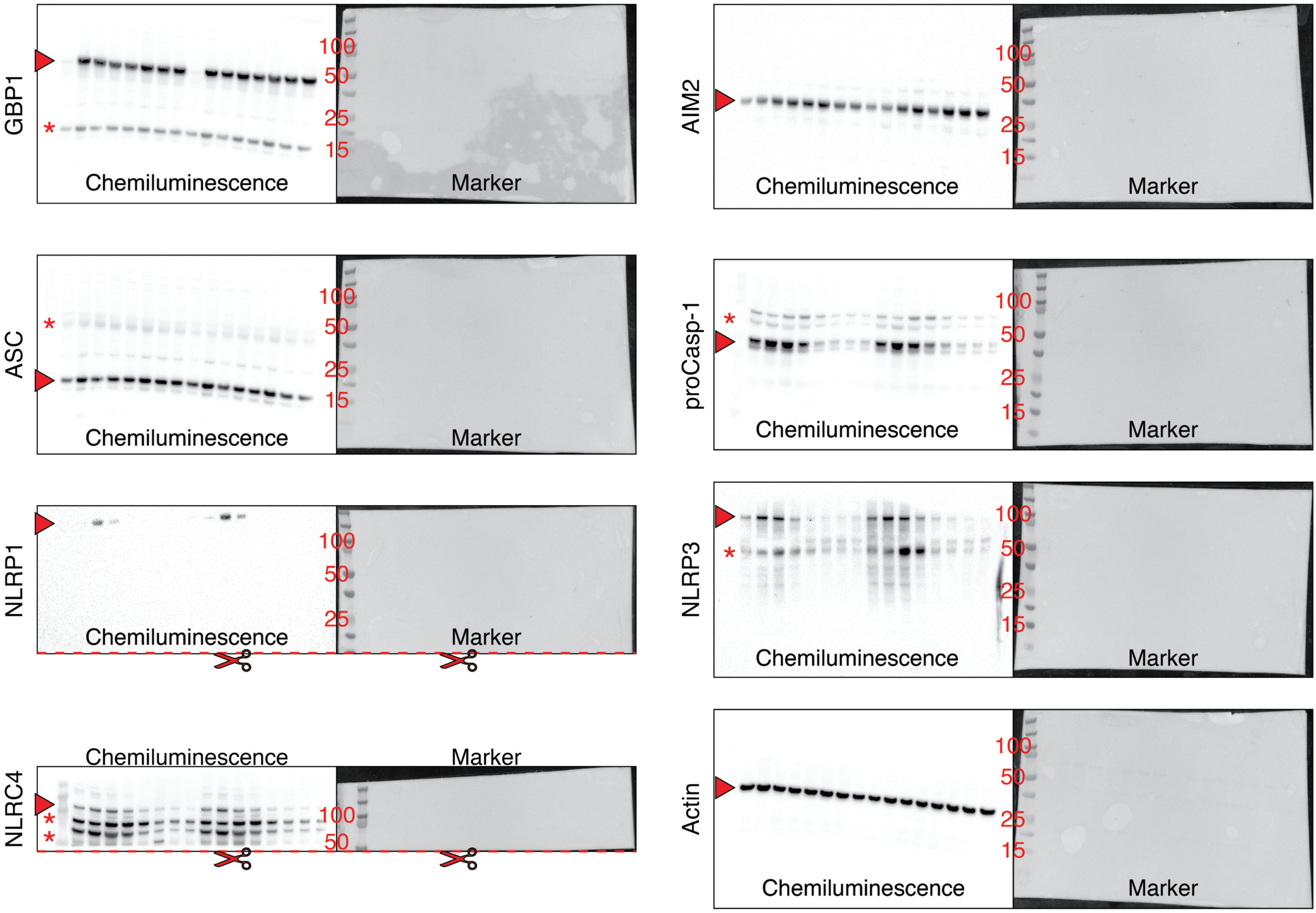

100 Size marker in kDa (Biorad, Precision Plus Dual color)

* Unspecific bands or bands from previous antibody

▶ Protein band of interest

✂ Indicates if membrane was cut prior to developing the immunoblot
