# Supplementary figures and images for: Human GBP1 is a microbe‐specific gatekeeper of macrophage apoptosis and pyroptosis
Source: EMBO J. 2019 Jun 3;38(13):e100926. doi: 10.15252/embj.2018100926 (PMC6600649; doi:10.15252/embj.2018100926)

# Immunoblots from Figure 6C

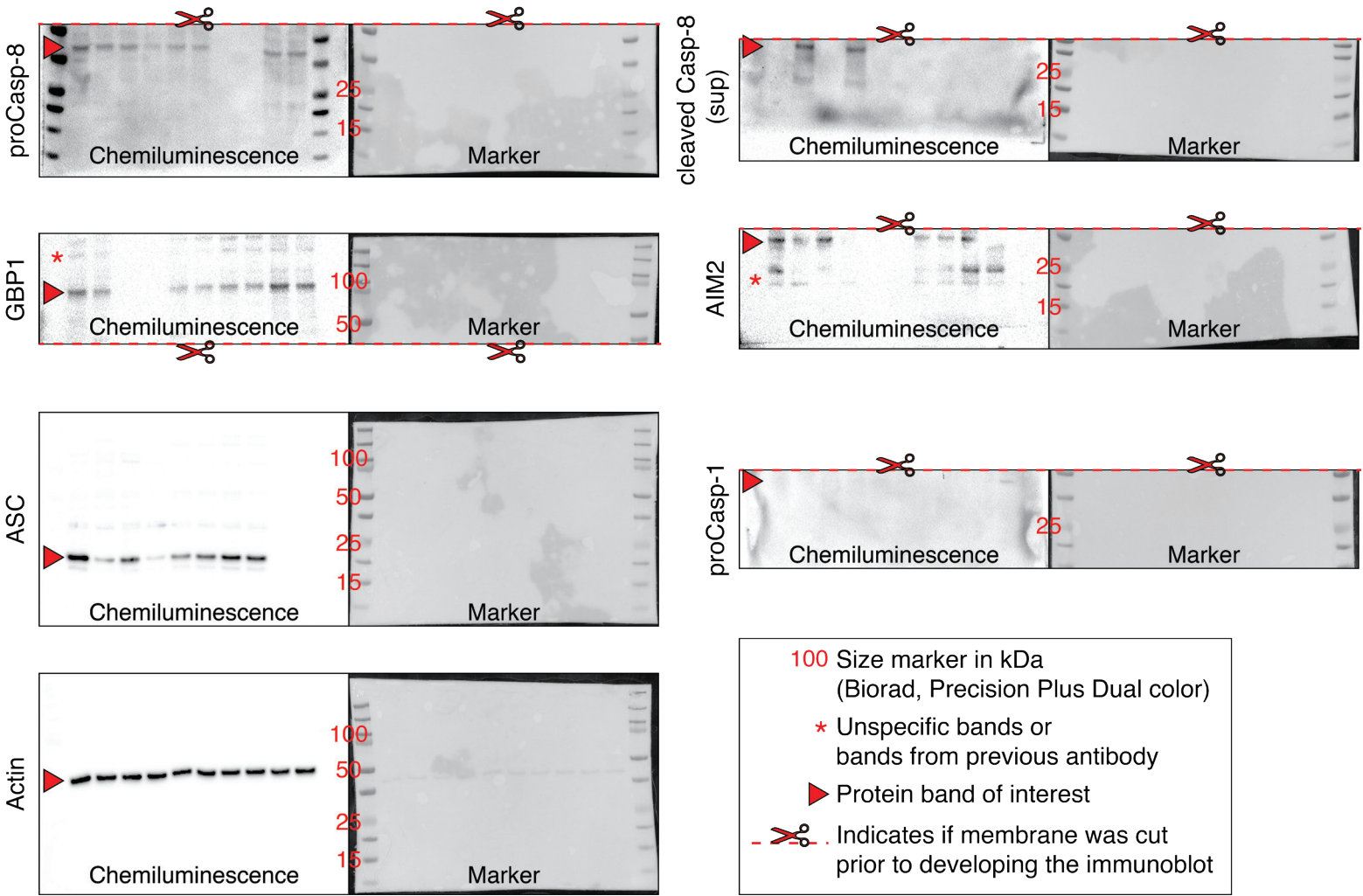

Supplement: Supplementary file 6 — Source Data for Figure 6 [file EMBJ-38-e100926-s005.pdf]
